# Supplementary material for: The pomegranate-derived peptide Pug-4 alleviates nontypeable Haemophilus influenzae-induced inflammation by suppressing NF-kB signaling and NLRP3 inflammasome activation
Source: PeerJ. 2024 Feb 20;12:e16938. doi: 10.7717/peerj.16938 (PMC10885808; doi:10.7717/peerj.16938)
Supplement: Supplemental Information 2 — Each blot was captured by CCD camera twice. In column A, exposure time on the Image Quant LAS4000 was set to 1/100 sec and images were captured in order that the molecular weight markers could be detected. The same blots were then captured by Image Quant LAS4000 with appropriate exposure times and the respective bands visualized (column B). [file peerj-12-16938-s002.pdf]

Original Western Blot Images

A pomegranate-derived peptide Pug-4 alleviates nontypeable Haemophilus influenzae-induced inflammation by suppressing NF-κB and NLRP3 inflammasome pathways

Pornpimon Jantaruk1[1], Sittiruk Roytrakul[2], Anchalee Sistayanarain[1] and Duangkamol Kunthalert[1, 3]*

[1] Department of Microbiology and Parasitology, Faculty of Medical Science, Naresuan University, Phitsanulok, Thailand

[2] National Center for Genetic Engineering and Biotechnology, National Science and Technology Development Agency, Thailand Science Park, Pathumthani, Thailand

[3] Centre of Excellence in Medical Biotechnology, Faculty of Medical Science, Naresuan University, Phitsanulok, Thailand

*Corresponding author:

Duangkamol Kunthalert

Email address: duangkamolk@nu.ac.th, kunthalertd@yahoo.com

Figure 5 Pug-4 peptide inhibited iNOS and COX-2 protein expression in NTHi-infected A549 cells.

Total proteins extraction

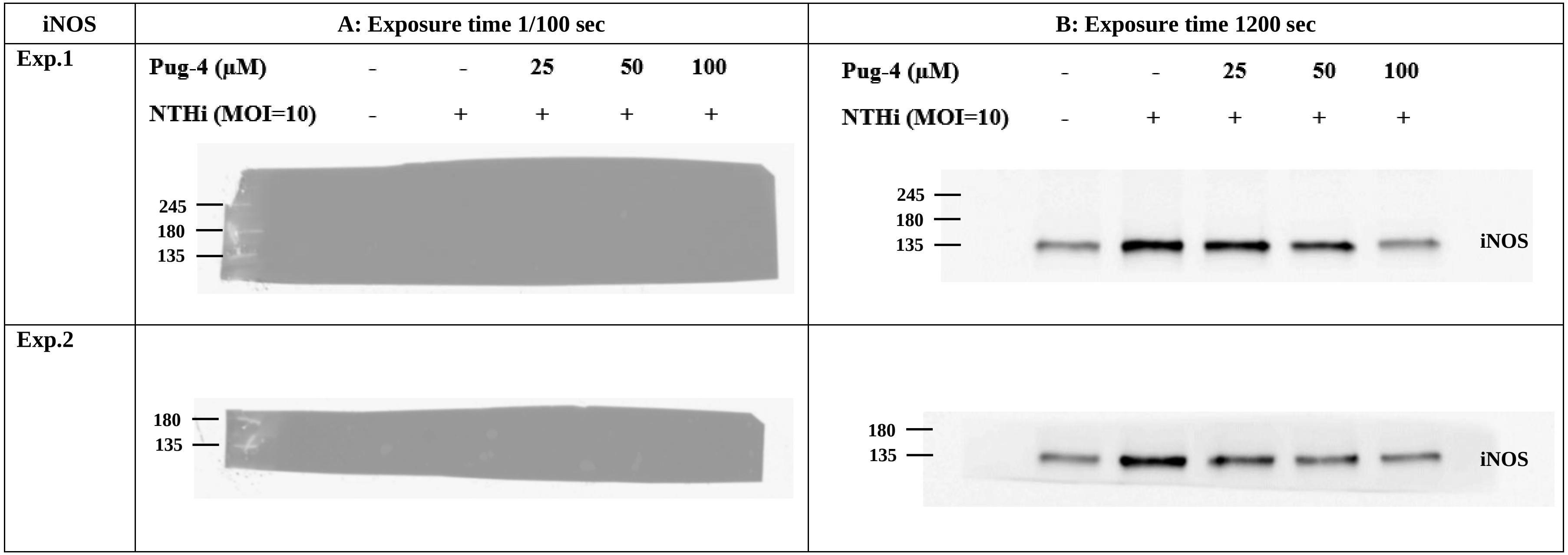

COX-2	A: Exposure time 1/100 sec	B: Exposure time 240 sec
Exp.1-1	Pug-4 (µM) - - 25 50 100<br>NTHi (MOI=10) - + + + +<br>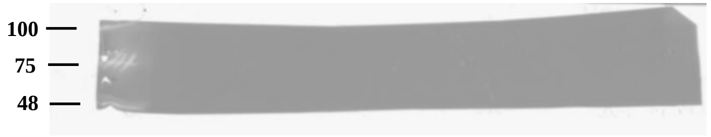	Pug-4 (µM) - - 25 50 100<br>NTHi (MOI=10) - + + + +<br>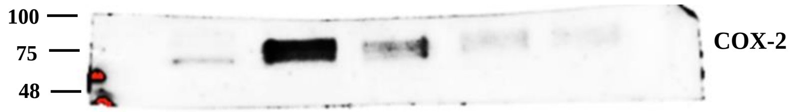
Exp.2-1	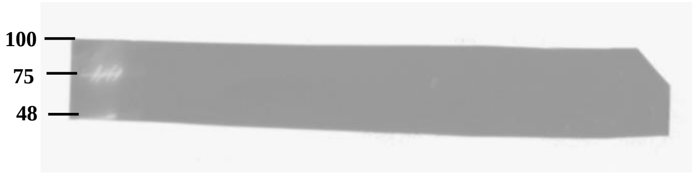	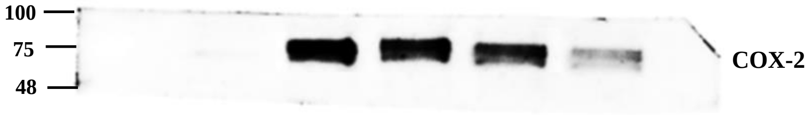
Exp.1-2	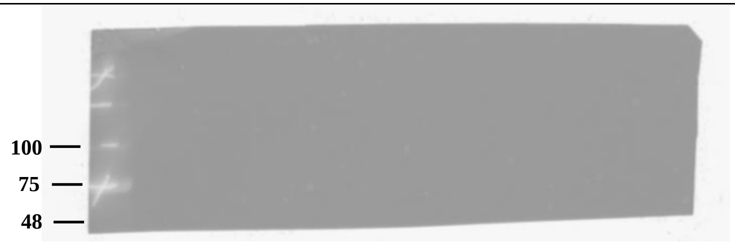	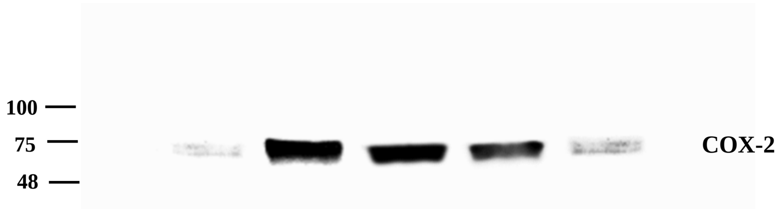
Exp.2-2	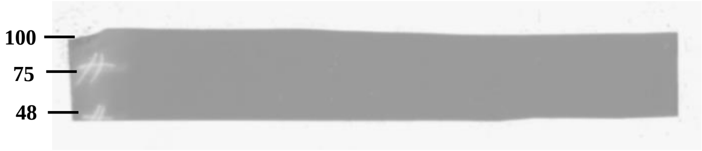	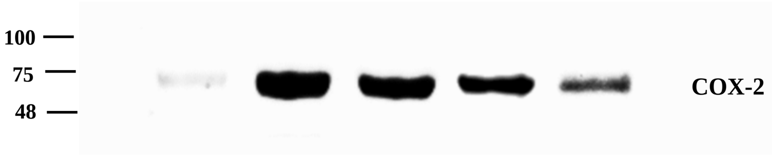

β-actin	A: Exposure time 1/100 sec	B: Exposure time 3 sec
Exp.1	Pug-4 (µM) - - 25 50 100<br>NTHi (MOI=10) - + + + +<br>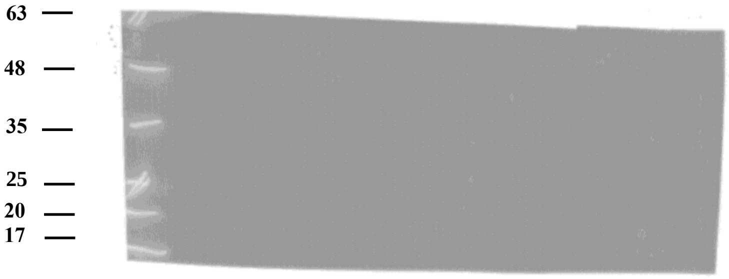	Pug-4 (µM) - - 25 50 100<br>NTHi (MOI=10) - + + + +<br>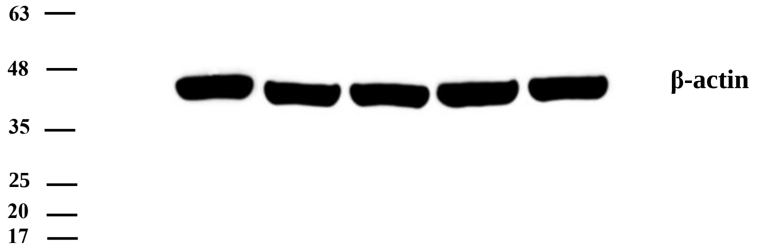
Exp.2	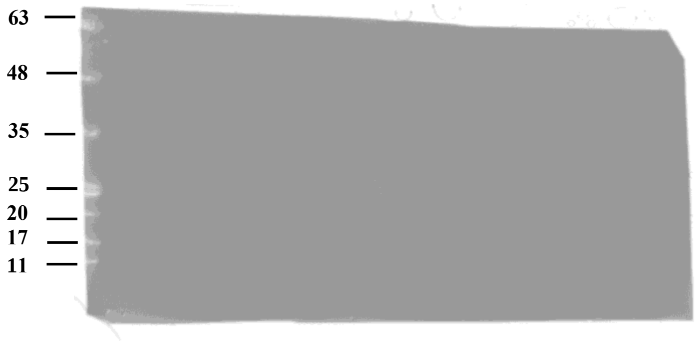	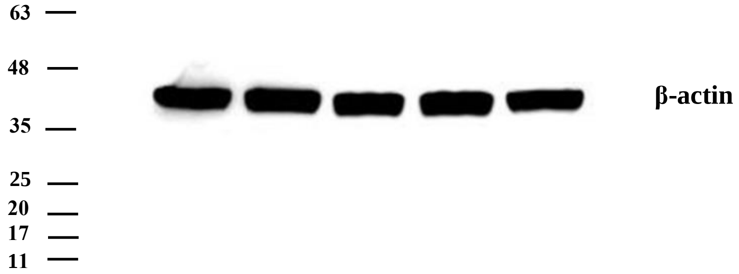

Figure 6 Pug-4 peptide inhibits NTHi-induced NF-κB activation in A549 cells.

Proteins from Nuclear extraction

<table>
<tr><th>NF-κB p65</th><th>A: Exposure time 1/100 sec</th><th>B: Exposure time 900 sec</th></tr>
<tr><td>Exp.1</td><td>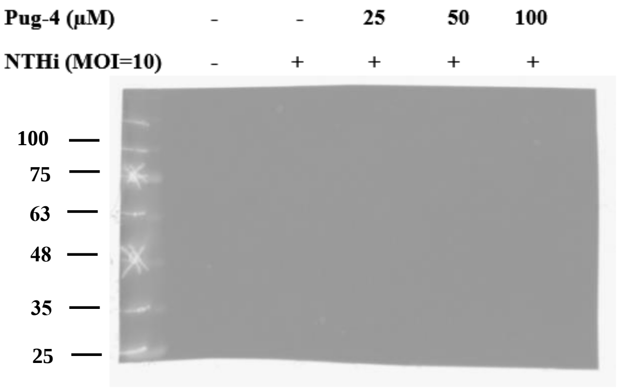
</td><td>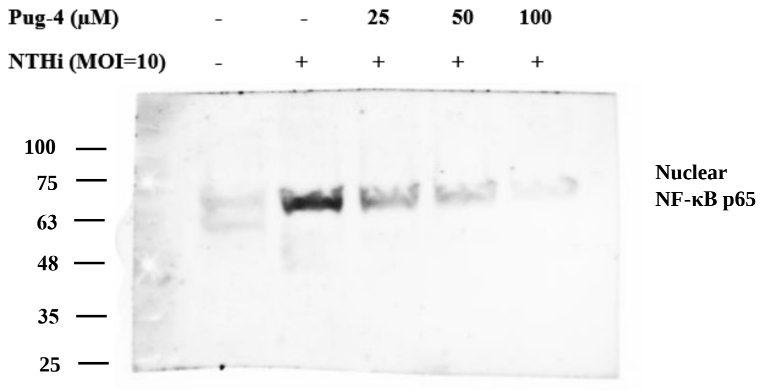
</td></tr>
<tr><td>Exp.2</td><td>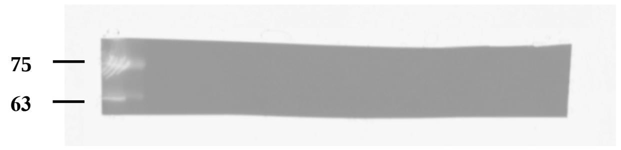
</td><td>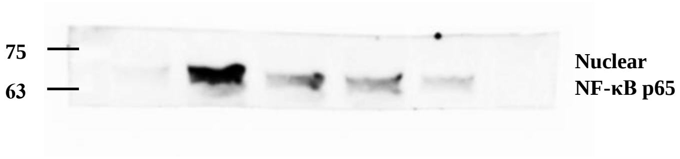
</td></tr>
</table>

Proteins from Cytoplasmic extraction

<table>
<tr><th>NF-κB p65</th><th>A: Exposure time 1/100 sec</th><th>B: Exposure time 240 sec</th></tr>
<tr><td>Exp.1</td><td>

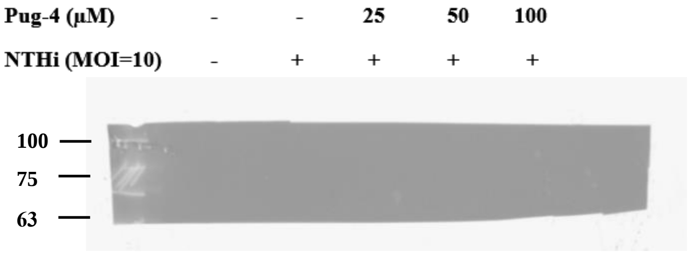

</td><td>

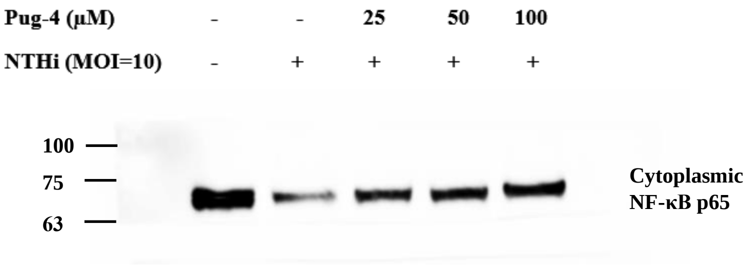

</td></tr>
<tr><td>Exp.2</td><td>

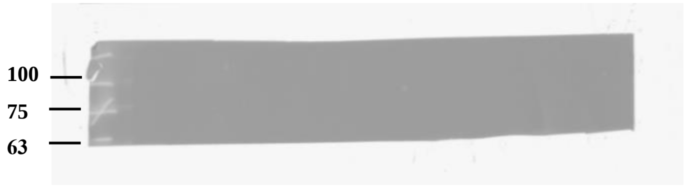

</td><td>

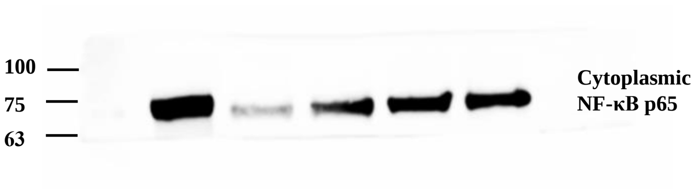

</td></tr>
</table>

β-actin	A: Exposure time 1/100 sec	B: Exposure time 3 sec
Exp.1	Pug-4 (µM) - - 25 50 100<br>NTHi (MOI=10) - + + + +<br>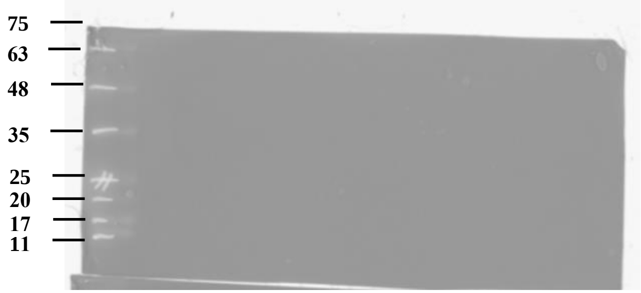	Pug-4 (µM) - - 25 50 100<br>NTHi (MOI=10) - + + + +<br>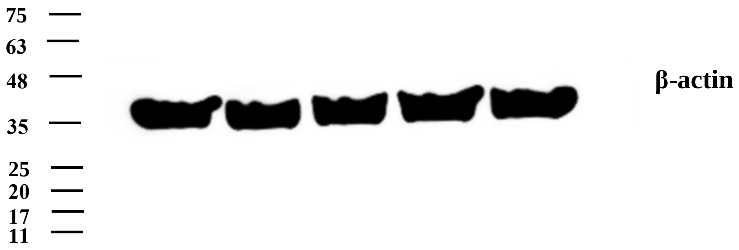
Exp.2	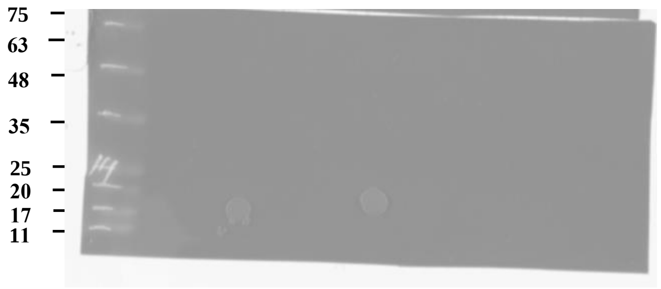	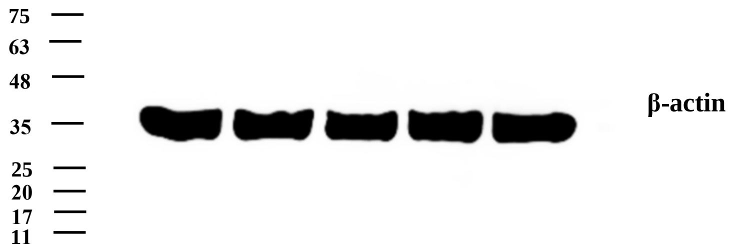

Figure 7 Pug-4 peptide suppressed NTHi-induced activation of NLRP3 inflammasome pathway in A549 cells.

Proteins from supernatant

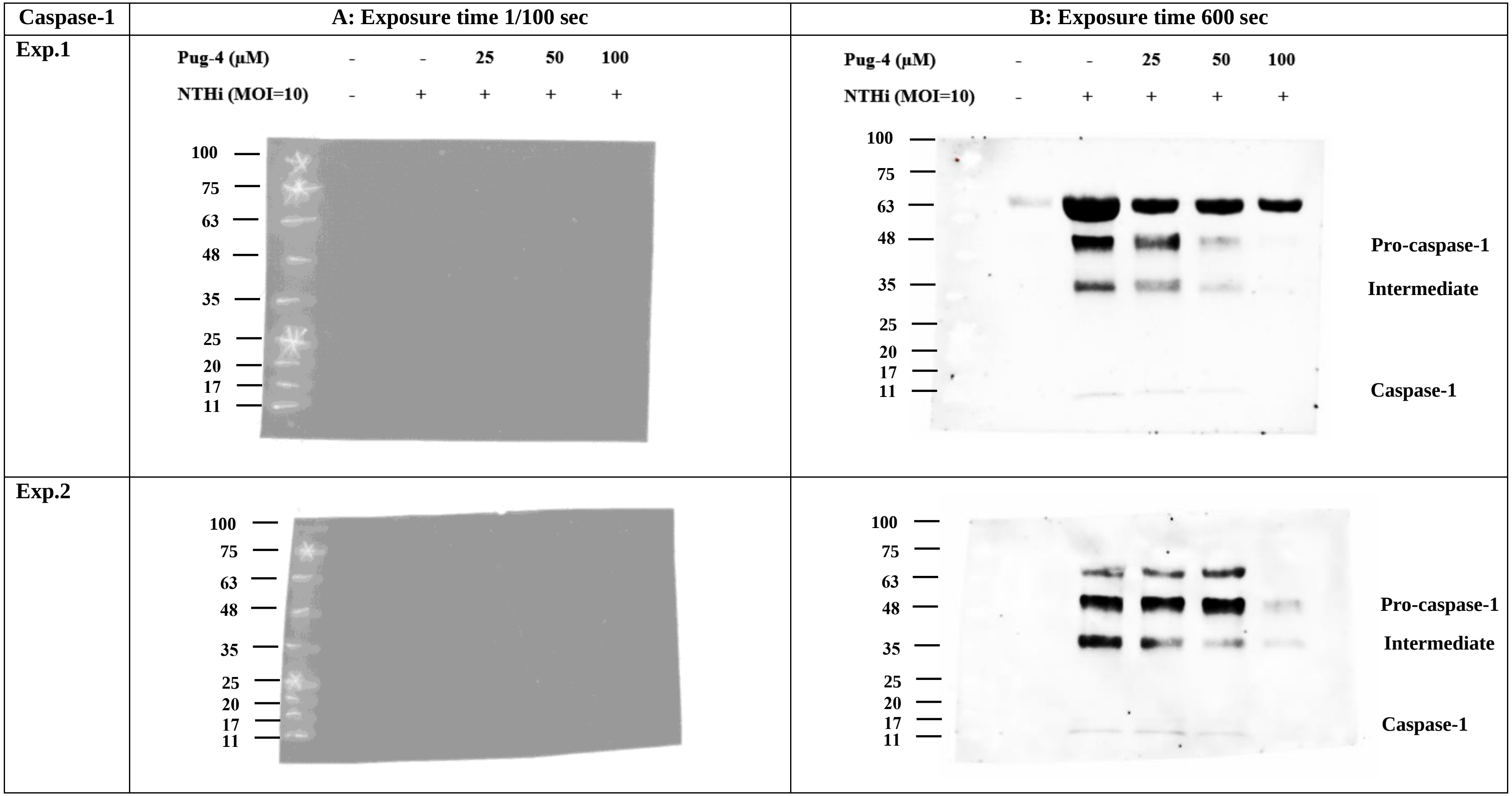

<table>
<tr><td>IL-1β</td><td>A: Exposure time 1/100 sec</td><td>B: Exposure time 600 sec</td></tr>
<tr><td>Exp.1</td><td>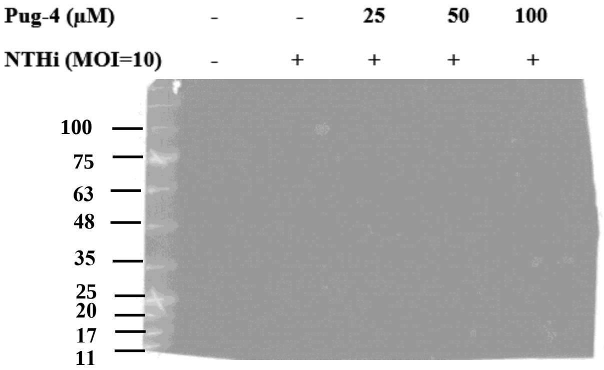
</td><td>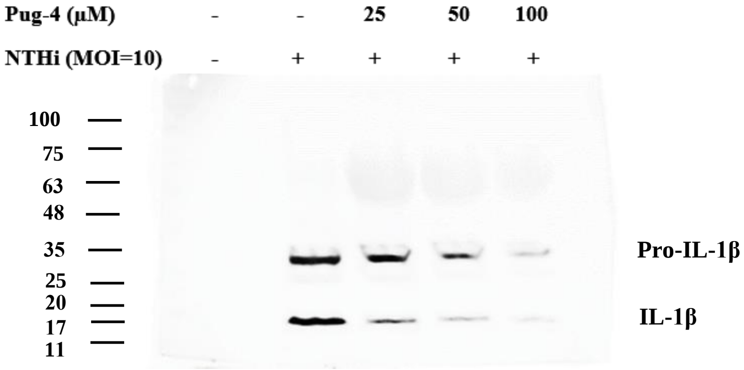
</td></tr>
<tr><td>Exp.2</td><td>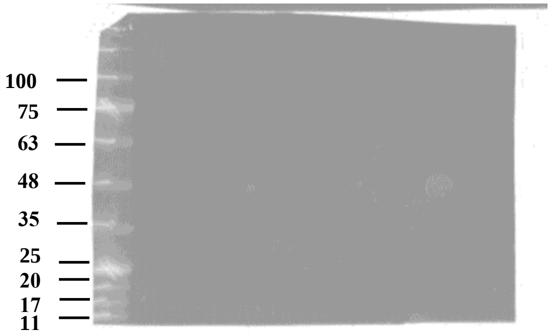
</td><td>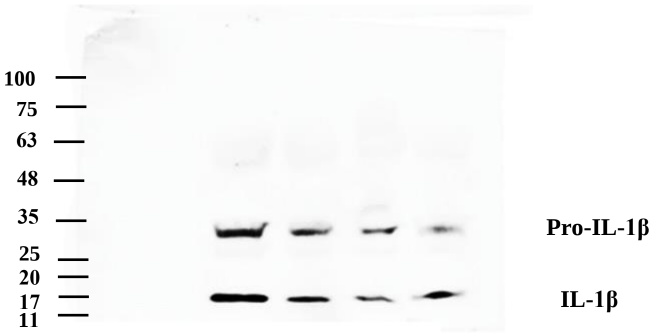
</td></tr>
</table>

Proteins from cell lysates

<table>
<tr><th>NLRP3</th><th>A: Exposure time 1/100 sec</th><th>B: Exposure time 240 sec</th></tr>
<tr><td>Exp.1</td><td>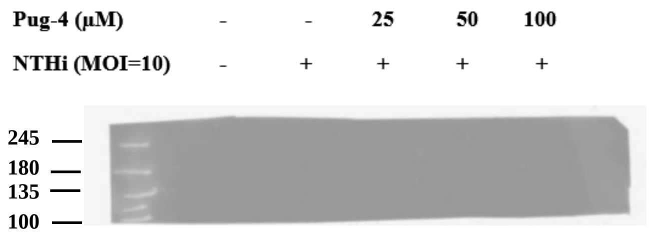
</td><td>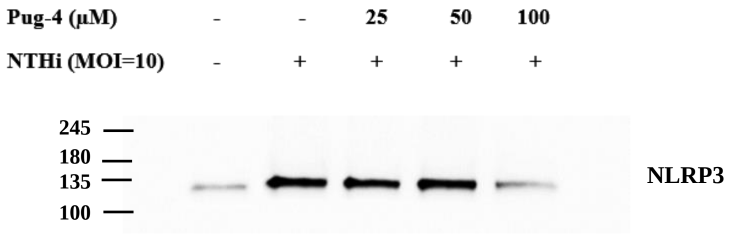
</td></tr>
<tr><td>Exp.2</td><td>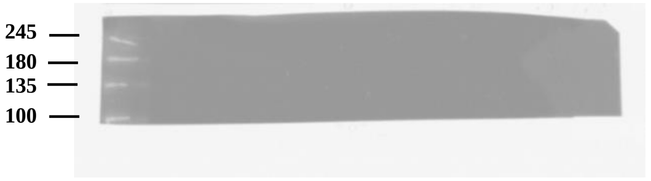
</td><td>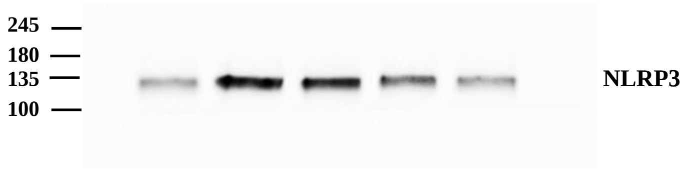
</td></tr>
</table>

β-actin	A: Exposure time 1/100 sec	B: Exposure time 3 sec
Exp.1	**Pug-4 (µM)** - - **25** **50** **100**<br>**NTHi (MOI=10)** - + + + +<br>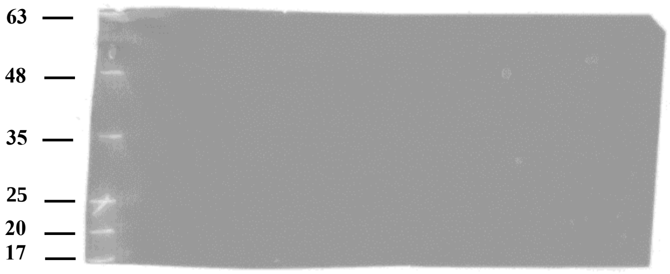	**Pug-4 (µM)** - - **25** **50** **100**<br>**NTHi (MOI=10)** - + + + +<br>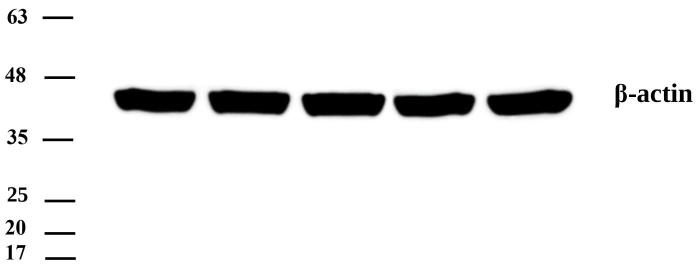
Exp.2	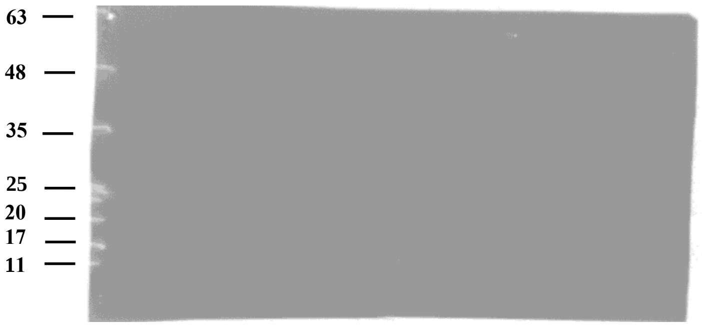	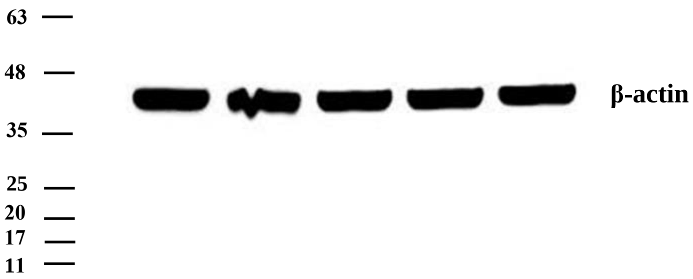
